# Individualized assessment of residual cognition in patients with disorders of consciousness

**DOI:** 10.1016/j.nicl.2020.102472

**Published:** 2020-10-20

**Authors:** Geoffrey Laforge, Laura E. Gonzalez-Lara, Adrian M. Owen, Bobby Stojanoski

**Affiliations:** a The Brain and Mind Institute, The University of Western Ontario, London ON N6A 5B7, Canada; b The Department of Psychology, The University of Western Ontario, London ON N6A 5B7, Canada; c The Department of Physiology and Pharmacology, The University of Western Ontario, London ON N6A 5B7, Canada

**Keywords:** Disorders of consciousness, Electroencephalography, Naturalistic stimuli, Components analysis

## Abstract

Patients diagnosed with disorders of consciousness show minimal or inconsistent behavioural evidence of conscious awareness. However, using functional neuroimaging, recent research in clinical neuroscience has identified a subpopulation of these patients who reliably produce neural markers indicative of awareness. In this study, we recorded electroencephalograms during a response-free movie task to assess narrative processing in patients with disorders of consciousness. Thirteen patients diagnosed with a disorder of consciousness and 28 healthy controls participated in this study. We designed a movie-watching/listening paradigm involving two suspenseful movie clips, one audiovisual and one audio-only, and used electroencephalography to extract patterns of brain activity that were maximally correlated between subjects. These activity patterns served as electrophysiological indices of narrative processing, which were compared to the neural responses of patients during the same movies. Our analysis revealed two patterns of neural activity, one for each movie condition, that were significantly and reliably correlated between healthy participants. Of the twelve patients who watched the audiovisual movie, 25% produced a pattern of activity that was significantly correlated with the healthy group, while of the ten who listened to the audio narrative, 30% produced electrophysiological patterns similar to controls (one patient responded appropriately to both). The method presented here allows for rapid bedside assessment of higher-order cognitive processing in patients with disorders of consciousness. By leveraging the common neural response to movie stimuli, we were able to identify comparable patterns of brain activity in individual, behaviourally non-responsive patients, reflecting a capacity for narrative processing.

## Highlights

• Single-trial electrical recordings index higher-order cognitive processing of movie stimuli.
• Common patterns of neural activity associated with the brain’s executive network.
• The time course of common neural activity correlates with ratings of suspense.
• 38% of non-responsive patients correlate with controls during movie-watching tasks.
• Novel bedside assessment of complex cognition in behaviourally non-responsive patients.

## 1 Introduction

A small but significant number of patients who survive severe brain injury will progress to a state of altered awareness known as a disorder of consciousness (DOC). Patients with DOC exhibit regular periods of wakefulness but produce minimal or inconsistent behavioural evidence of conscious awareness. This presents a considerable challenge for clinicians when trying to accurately diagnose a patient’s conscious state, as available clinical measures like the Coma Recovery Scale-Revised (CRS-R; Kalmar and Giacino, 2005) rely on observable behavioural responses to verbal commands. In some cases, a lack of purposeful behaviour may reflect a true absence of awareness—a condition known as the vegetative state. However, expert reassessments of DOC patients consistently show that approximately 40% of these patients are, at least, minimally conscious (Andrews et al., 1996, Burke, 2002, Childs et al., 1993, Schnakers et al., 2009). While repeated behavioural assessments may reduce the rate of misdiagnosis of patients with DOC, acquired cognitive or physiological impairments may still preclude behavioural expressions of awareness in many patients. Because of these limitations, novel brain-based assessments have been proposed as an alternative to behavioural testing.

To date, several studies have demonstrated that neuroimaging techniques, such as functional MRI (fMRI) and electroencephalography (EEG), can be used to capture the neural correlates of awareness in patients with DOC. For example, Owen et al. (2006) developed an fMRI motor imagery task to assess “covert” (rather than behavioural) command-following. In that study, the unique patterns of brain activity elicited by different types of imagined motor imagery (e.g., playing tennis, spatial navigation) were used to determine whether patients could correctly modulate their neural activity in response to specific task instructions. One patient, who appeared to be entirely vegetative, could reliably produce the appropriate neural response to each imagery command, providing strong evidence of her awareness. Similar imagery paradigms have since been used to examine larger cohorts of patients using either fMRI (Monti et al., 2010) and, more recently, EEG (Cruse et al., 2011). Yet, the active nature of imagined command-following tasks, much like their behavioural counterparts, requires the coordination of several cognitive faculties, as well as sustained periods of vigilance and effort, that may prove difficult for some patients and impossible for others. Indeed, a recent review found that just 14% of behaviourally non-responsive patients could modulate their brain activity in response to verbal commands (Kondziella et al., 2016), which is far lower than the estimated 40% who are known to be misdiagnosed (Andrews et al., 1996, Childs et al., 1993, Schnakers et al., 2009).

As a result, recent studies have moved towards using naturalistic tasks that more closely mimic real-world activities. Movie-watching has emerged as a particularly useful paradigm; previous research has shown that watching suspenseful movies such as Alfred Hitchcock’s “*Bang! You’re Dead*” produces significant brain-wide correlations between healthy controls (Hasson et al., 2010, Hasson et al., 2004, Naci et al., 2015). This “synchronization” spans primary sensory regions as well as areas of the frontal and parietal cortices that are involved in executive functions like theory of mind and attentional control (Naci et al., 2015, Naci et al., 2014), both of which are necessary to follow the plot of a movie. Naci and colleagues (2014) capitalized on this phenomenon to create an fMRI movie-watching paradigm for assessing executive processing in patients with DOC. They showed that the degree of frontoparietal synchronization between participants during “*Bang! You’re Dead*” significantly correlated with measures of suspense and executive load. Furthermore, the same highly-correlated brain responses occurred in one patient who met the behavioural criteria for a vegetative state diagnosis. On this basis, the authors were able to conclude that the patient was, in fact, aware, despite his behavioural and clinical profile.

However, for naturalistic approaches to be clinically viable, they must be moved to the bedside. In this regard, EEG is the ideal neuroimaging tool for assessing residual cognitive function in patients with DOC; EEG is portable, widely available in clinical settings, and it minimizes the cost of routine neural assessments, as well as the physical toll incurred by patients during fMRI testing (Cruse et al., 2011). To this end, we hypothesized that EEG could be used to assess the level of inter-subject synchronization (or inter-subject correlations; ISCs), and therefore identify markers of executive processing in patients with DOC. As such, the aim of this study was to develop a bedside neuroimaging paradigm to assess ISCs during movie tasks in patients with DOC.

## 2 Materials and methods

### 2.1 Patients and controls

We recruited a convenience sample of 13 patients with severe traumatic and non-traumatic brain injuries who met the CRS-R (Kalmar and Giacino, 2005) diagnostic criteria for DOC (see Table 1 for clinical information). At the time of testing, ten patients met the clinical criteria for the vegetative state, two were in a minimally conscious state, and one was diagnosed with Locked-in Syndrome. Informed assent was obtained from substitute decision-makers and medical care teams for all patients. All healthy participants were recruited from The Brain and Mind Institute at the University of Western Ontario, Canada. Twenty-eight healthy volunteers took part in the EEG portion of this study, and an additional 40 performed a follow-up behavioural task. Informed written consent was acquired prior to testing.

**Table 1:** 

	**Age at Assessment (years)**	**Sex**	**ClinicalDiagnosis**	**Etiology**	**Interval postictus (days)**	**CRS-R at Assessment (/23)**	**Movie Condition(s)**	**Significant ISC**
Patient 1	27	Male	VS	TBI	3647	6	Both	TKN*
Patient 2	41	Male	VS	Anoxia	1148	7	Both	–
Patient 3	51	Male	LIS	Stroke	1934	15	Both	TKN*
Patient 4	38	Male	VS	Anoxia	7058	6	TKN	–
Patient 5	48	Female	VS	TBI	8427	5	Both	BYD*
Patient 6	60	Male	VS	Anoxia	2463	3	Both	–
Patient 7	29	Female	MCS	TBI	3252	8	Both	–
Patient 8	21	Male	VS	TBI	1349	2	Both	–
Patient 9	15	Female	VS	Anoxia	1072	6	Both	–
Patient 10	52	Female	VS	Anoxia	3592	5	Both	**
Patient 11	25	Male	VS	TBI	1198	5	BYD	BYD*
Patient 12	63	Male	MCS	Anoxia	368	9	BYD	–
Patient 13	19	Male	VS	Anoxia	314	6	BYD	–

*Note*. VS, Vegetative State; MCS, Minimally Conscious State; LIS, Locked-in Syndrome; TBI, traumatic brain injury; CRS-R, Coma Recovery Scale-Revised; BYD, “*Bang! You’re Dead*”; TKN, “*Taken*”.

*denotes significant ISC with controls, *p* < 0.05; ** denotes significant ISC with controls for both movie tasks, *p* < 0.05.

Ethics approval for this study was granted by the Health Sciences Research Ethics Board and the Non-Medical Research Ethics Board of The University of Western Ontario.

### 2.2 Procedures

All patients were assessed with the CRS-R on the day of testing. The CRS-R consists of six subscales evaluating sensory and motor function, communication ability, and level of arousal, to distinguish patients who are minimally conscious—those who exhibit intermittent behavioural evidence of awareness—from patients who are in a vegetative state (Kalmar and Giacino, 2005).

We used two suspenseful movie clips to measure ISCs between healthy controls and individual patients with DOC. The first clip was an 8-minute audiovisual segment from the Alfred Hitchcock TV movie “*Bang! You’re Dead*”. Briefly, this scene portrays a 5-year-old boy who finds his uncle’s revolver. Being unaware of its danger, the boy partially loads the gun and plays with it as if it were a normal toy. The viewer (and the boy himself) is rarely privy to whether the gun has a bullet in its chamber, and suspense continues to build the longer the boy plays with the gun (e.g., spinning the chamber, pointing it at others, pulling the trigger). To account for potential visual impairments among DOC patients, we also used a second clip comprised of a 5-minute audio excerpt from the movie “*Taken*. In this clip, the listener hears a phone conversation between a father character and his daughter, who is away on vacation. The conversation quickly changes tone as she becomes aware of kidnappers in her accommodation. The kidnappers eventually discover where she is hiding and take her away—all of which can be heard over the father’s end of the call. Unlike “*Bang! You’re Dead*”, the suspense in this clip builds much less subtly, relying more on atmosphere and intensity than unpredictability. This brute-force approach to building suspense was taken into account when initially testing this clip (Naci et al., 2015), since driving synchronization with audio alone is more difficult than with visual or multimodal stimuli (Dmochowski et al., 2017, Naci et al., 2015). Both movies have been rated as highly suspenseful and produce robust ISCs between healthy volunteers in fMRI (Naci et al., 2015, Naci et al., 2014). We also used two “scrambled” control stimuli, one for each movie, to separate the neural responses elicited by the sensory properties of watching or listening to the movies from those involved in following the plot. The scrambled version of “*Bang! You’re Dead*” was generated by isolating 1 s segments of the movie and arranging them in a pseudorandom order, thereby eliminating the temporal coherence of the narrative (Naci et al., 2014). To create the scrambled version of “*Taken*”, the audio was spectrally rotated, which preserved many of its acoustic features but rendered the speech indecipherable (Naci et al., 2015). The scrambled movie clips were presented before the intact versions for all patients and participants to prevent potential carry-over effects of the narrative.

Two separate groups of healthy volunteers were recruited for this study: 13 participants watched the intact and scrambled versions of “*Bang! You’re Dead*”, and 15 participants heard both versions of “*Taken*”. Individual participants were seated in a dimly lit room and instructed to watch or listen attentively to the stimuli. The task instructions and design remained the same when testing patients with DOC. Each patient was presented with one or both movie types (12 “*Bang! You’re Dead*”; 10 “*Taken*”; 9 both) with the presentation order counterbalanced between patients.

Stimulus presentation was controlled with the Psychtoolbox plugin for Matlab (Brainard, 1997, Kleiner et al., 2007, Pelli, 1997) on a 15″ Apple MacBook Pro. The laptop screen was used to present the video component of “*Bang! You’re Dead*” but remained blank (black) during “*Taken*”. All audio was presented binaurally to participants at a comfortable listening volume through Etymotics ER-1 in-ear headphones. The EEG data were analyzed using EEGLAB software (Delorme and Makeig, 2004). The data were cleaned following standard preprocessing steps including re-referencing, filtering, and removal of artifacts (e.g., ocular, motor). Finally, estimates of cortical activity during “*Taken*” were computed with the Brainstorm software for MATLAB (Tadel et al., 2011). Source reconstructions were performed only for “*Taken*” because of the availability of T1 structural MRI scans among participants in this condition.

### 2.3 EEG acquisition

EEG data were collected using a 129-channel cap (Electrical Geodesics Inc. [EGI], Oregon, USA). Signals were sampled at 250 Hz and referenced online to the vertex (Cz). Electrode impedances were kept below 50kΩ. Offline processing was performed using MATLAB software, including custom scripts and the EEGlab toolbox (Delorme and Makeig, 2004). Offline, the EEG data were re-referenced to the common average and bandpass filtered from 0.5 − 60 Hz (notch at 60 Hz). Automatic artifact detection (EEGLAB) was used to identify bad channels, which were removed, then interpolated back into the data. We then used an independent components analysis (ICA) to visually identify patterns of neural activity characteristic of eye and muscle movements which were removed from the data. The data were also de-spiked to reduce the influence of aberrant peak amplitudes on further analyses (Dmochowski et al., 2012). EEG preprocessing was performed separately for each participant, movie, and stimulus condition.

### 2.4 Statistical analyses

We performed a correlated components analysis (CorrCA; Dmochowski et al., 2012, Ki et al., 2016) to calculate ISCs from the EEG data. CorrCA identifies linear combinations of stable and distinct patterns of brain activity to generate “components” that are maximally correlated (using Pearson’s rho) between participants (see Cohen and Parra, 2016, Ki et al., 2016 for calculations). Here, the components serve a similar purpose to those extracted from fMRI data using group-level ICA, in that they reflect common patterns of neural activity across participants. Since components derived by the CorrCA are rank-ordered by the magnitude of their correlations, we focused on the top-ranked component for each movie condition.

A CorrCA was first computed in healthy controls for each movie (“*Bang! You’re Dead*” and *“Taken”*) and condition (intact, scrambled). In computing the CorrCA (Dmochowski et al., 2012, Ki et al., 2016), the spatial weights of the top component are back-projected onto the EEG recordings from individual participants, creating a spatial filter of the data, which isolates the underlying signal of the component and its activity over time. These per-subject component time courses are then correlated between all pairs of participants, and the mean of the pairwise correlations for each individual participant represents their overall ISCs; that is, how “synchronized” each participant is to the group as a whole.

Leave-one-out cross-validation and permutation testing were then used to determine the reliability of the components as well as evaluate the statistical significance of individual ISCs during each movie. The leave-one-out approach involved iteratively removing one participant from the group and recomputing the CorrCA (which generated new components), and the extracted time courses for each iteration of the CorrCA were later used to compute ISCs. That is, we repeated the CorrCA 13 times for “*Bang! You’re Dead*” and 15 times for “*Taken*”—leaving out a different participant during each recalculation–and computed ISCs between the left-out participant and the set of participants included in each iteration of the CorrCA. This also enabled us to compare the components topographies generated by the CorrCA across subsets of the group and measure the average degree of synchronization for each participant across these subsets. This approach ensured that the components extracted using CorrCA and the subsequent ISCs between participants were unbiased and reliable.

Permutation testing was then used to establish thresholds of statistical significance for the ISCs of individual participants. This was done by phase-shifting the correlation coefficients between participants (Dmochowski et al., 2012, Ki et al., 2016, Theiler et al., 1992) and performing a 1000 iteration resampling procedure to create individual null distributions. The top 5% of the distributions formed the significance thresholds for each participant (*p* < 0.05 FDR corrected). The leave-one-out and permutation analysis also served as a statistical benchmark for assessing the extent to which individual DOC patients were synchronized to healthy controls during the movies. The analysis followed a similar procedure with one exception: rather than computing new CorrCA components using patient data, we back-projected the initial components from healthy controls onto their EEG. In this way, we could directly compare the neural activity from patients to the healthy group.

### 2.5 Suspense ratings and temporal Inter-subject correlations

To verify that the component extracted by the CorrCA represented neural activity associated with executive processing of the plot (Dmochowski et al., 2012, Cohen and Parra, 2016, Poulsen et al., 2017), we examined whether the temporal fluctuations of ISCs coincided with subjective ratings of suspense during both movies. To do this, we first collected suspense ratings for “*Bang! You’re Dead*” and “*Taken*” from two independent samples of 20 healthy volunteers. Participants rated how much “suspense” they felt at 2 s intervals throughout the movie, ranging from 1 (least) to 10 (most). Individual ratings were then averaged to create a group-level time course of suspense ratings specific to each movie. Second, we used a sliding window technique—set at 2 s intervals to align with the sampling frequency of the suspense ratings—to identify time periods when the EEG activity from each participant was significantly correlated to the mean of the group (based on a leave-one-out approach). Significance was established against null distributions that were generated for each participant at every time window (2 s) throughout the movie by randomly shuffling (using phase-shifting; Dmochowski et al., 2017, Ki et al., 2016, Theiler et al., 1992) the component time course, recomputing the ISCs 1000 times, and retaining the value that corresponded to the 95th percentile. ISCs that exceeded this threshold at each time point were considered statistically significant. Group-level temporal ISCs were then calculated by summing the number of participants who were significantly synchronized to the group at every time point. Finally, we correlated the time course of the significant group-level temporal ISCs for each movie and condition (intact and scrambled versions) to their corresponding suspense ratings using both frequentist and Bayesian statistics.

### 2.6 Component source modelling

For those participants who listened to the “*Taken*” clip, we performed an exploratory source localization analysis using Brainstorm (Tadel et al., 2011) and a spatiotemporal regression (Custo et al., 2014) to uncover the potential cortical sources of the components. Head and cortical models were constructed using T1 weighted structural MRI images and automatic (OpenMEEG) boundary-element modelling (Gramfort et al., 2010, Mosher et al., 1999). To improve the accuracy of the source estimates, electrode placements were captured for each participant during EEG acquisition using EGI’s Geodesic Photogrammetry System and co-registered to their corresponding head models. Sources were reconstructed from full EEG recordings from healthy controls for the intact and scrambled versions of *“Taken”* using a Tikhonov-regularized weighted minimum norm estimate with normalized current density maps. Individual cortical models and source estimates were then normalized to MNI (Montreal Neurological Institute) standard space. A spatiotemporal regression analysis (Custo et al., 2014) was performed to identify cortical sources that correlate with the group-level component time courses for each version of “*Taken*”. We then repeated the regression using the auditory envelope of the stimulus. Significant beta maps (corrected for multiple comparisons) were exported to SPM (Statistical Parametric Mapping), where we computed group-level *t* contrasts between the intact (intact > scrambled) and scrambled (intact < scrambled) audio conditions. This yielded contrast maps of the significant differences in functional activity associated with the activity of the top components from the CorrCA.

## 3 Results

### 3.1 Neural synchronization during naturalistic audiovisual stimulation

**Fig. 1:**
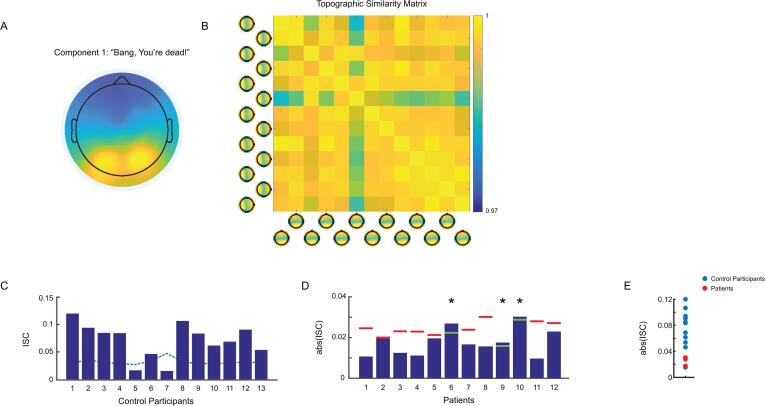
Component topographies and inter-subject correlations during “*Bang! You’re Dead*”. **A)** The spatial weights that maximize Pearson’s correlation (*r*) between healthy controls during the intact version of “*Bang! You’re Dead*”. **B)** The similarity matrix and polarity-normalized component topographies computed from iterative leave-one-out recalculations of the CorrCA. Spatial correlations are plotted across the scalp topographies, rather than the typical voltage mappings. Warmer colours indicate higher *r* values. **C)** Mean inter-subject correlations between healthy controls during the intact version of “*Bang! You’re Dead*”. Statistical thresholds (blue dashes) were calculated on a per-subject basis using a permutation test approach. **D)** Mean inter-subject correlations between individual patients and the healthy control group during the intact version of “*Bang! You’re Dead*”. Statistical thresholds (red/green) were determined on an individual basis for each patient using a permutation approach. Green thresholds and asterisks denote significance at *p* < 0.05 (see SI Fig. 5 A for raw patient ISCs with controls). **E)** The distribution of ISCs for control participants (blue) and three patients who were significantly correlated to the healthy group (red) during “*Bang! You’re Dead”*. (For interpretation of the references to colour in this figure legend, the reader is referred to the web version of this article.)

For the intact version of “*Bang! You’re Dead*”, the CorrCA produced a component topography that showed extensive frontal negativity and widespread posterior positivity among healthy controls (Fig. 1A). This component was remarkably reliable between smaller subsets of control participants (spatial correlations, r > 0.95; Fig. 1B), as demonstrated by the leave-one-out recalculations of the CorrCA. In effect, we found nearly identical patterns of neural activity each time we performed the CorrCA, irrespective of the participants included in the analysis; the group-level component for “*Bang! You’re Dead*” was not simply the product of the specific configuration of our sample but, rather, captured the most common neural response to watching this movie. The ISCs, likewise, showed a similar degree of reliability. At the group level, the mean ISCs during “*Bang! You’re Dead*’ (*M* = 0.084, *SD* = 0.053) were significant, *t*(12) = 5.700, *p* = 9.98e-5, confirming both that our task was generating inter-subject synchronization and that our EEG analyses could identify this synchrony. In fact, between individual participants, we found 85% whose EEG activity was significantly correlated to the rest of the group during “*Bang! You’re Dead*” (*p* < 0.05 FDR corrected; Fig. 1C).

At the group level, the temporal ISCs showed a comparable degree of consistency. We found that the EEG from healthy controls were significantly synchronized at the same time points for 25.32% of the intact version of “*Bang! You’re Dead*” (based on permutation statistics). Moreover, the group-level synchronization was significantly correlated to the average suspense ratings throughout the movie, *r* = 0.179, *p* = 6.00e-3, BF_10_ = 3.541. This revealed that individuals’ neural activity was most synchronized at times when the movie was most suspenseful, which suggested, therefore, that the top CorrCA component reflected executive processing of the movie (Cochen et al., 2017, Naci et al., 2014).

We then performed the CorrCA on the EEG data from the same 13 healthy controls during the scrambled version of “*Bang! You’re Dead*” to compare the degree of ISCs to the intact condition. The CorrCA produced a component that closely resembled the intact condition and was equally consistent across leave-one-out subsets (spatial correlations, *r* > 0.95). The group-level mean ISCs (*M* = 0.071, *SD* = 0.032) remained significant during the scrambled movie, *t*(12) = 8.047, *p* = 3.54e-6. While this corresponded to previous fMRI studies (Naci et al., 2015, Naci et al., 2014), we tested whether the components calculated for each condition reflected similar underlying neural processes. To do this, we back-projected the intact and scrambled components onto the EEG data from the other movie condition (intact onto scrambled and scrambled onto intact) and recomputed the ISCs. This produced a unique series of ISCs representing the overlap in neural processes captured by the components in both movie conditions. We predicted that if the components encompassed the same neural processes, the magnitude of the ISCs would remain largely unchanged. However, this was not the case. At the group level, we observed a significant reduction in group-level mean ISCs for both the intact, *t*(12) = 3.640, *p* = 3.00e-3, and scrambled movie conditions, *t*(12) = 2.659, *p* = 2.10e-2, which confirmed that, despite displaying similar levels of synchronization, the intact and scrambled components did not arise from the same underlying processes. As a follow-up, we ran a 2x2 factorial ANOVA to ensure that this effect was not driven by an interaction between the different movie conditions and the component projection type (i.e., correct or incorrect). The ANOVA confirmed a main effect of projection type, *F*(1,12) = 20.83, *p* = 7.00e-4 but did not reveal a significant condition by projection type interaction, *F*(1,12) = 0.73, *p* = 4.1e-1 (SI Fig. 1A).

The temporal ISCs revealed that, during the scrambled version of “*Bang! You’re Dead*”, participants’ EEG activity was significantly synchronized for 20.25% of the movie—5% less than the intact version (SI Fig. 2 A, B). Although this reduction in significant temporal ISCs was markedly less pronounced than those reported by Dmochowski et al. (2012), the temporal ISCs for this condition did not correlate with the suspense ratings for the intact movie, *r* = 0.045, *p* = 4.86e-1, BF_10_ = 0.103. What this suggests is that, although participants EEG activity was still synchronized to a comparable degree during the scrambled version of the movie, this was unrelated to the underlying elements of the plot, like its suspense (Naci et al., 2014).

**Fig. 2:**
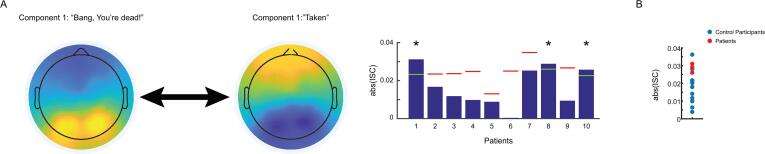
Component topographies for both movie conditions and inter-subject synchronization between patients and controls during “*Taken*”. **A)** Maximally correlated components calculated between healthy controls during the intact versions of “*Bang! You’re Dead*” (left) and “*Taken*” (middle), shown for comparison. Mean inter-subject correlations between individual patients and the healthy control group during the intact version of “*Taken*”. Statistical thresholds (red/green) were determined on an individual basis for each patient using a permutation approach. Green thresholds and asterisks denote significance at *p* < 0.05. **B)** The distribution of ISCs for control participants (blue) and three patients who were significantly correlated to the control group (red) during “*Taken*” (see SI Fig. 5B for raw patient ISCs with controls). (For interpretation of the references to colour in this figure legend, the reader is referred to the web version of this article.)

Using the component from the intact movie condition, we then calculated ISCs for 12 DOC patients while they watched the intact version of “*Bang! You’re Dead*”. Overall, 25% of patients had EEG activity that was significantly correlated with healthy controls’ during this movie (*p* < 0.05 FDR corrected; Fig. 1D), though the magnitude of their absolute correlations with controls was markedly lower on average. Notably, all of these patients met the behavioural criteria for a vegetative diagnosis at the time of testing. We repeated this procedure using the component topography for the scrambled version of the movie. Scrambled data were available for 10 of the 12 patients who watched “*Bang! You’re Dead!*”. During the scrambled movie condition, 20% of individual DOC patients’ neural activity was significantly synchronized with controls (*p* < 0.05 FDR corrected), though these were not the same patients who were significantly synchronized to the control group during the intact movie. While there were a similar number of patients whose neural activity was significantly correlated with controls’ during either the intact or scrambled condition, the majority of patients (70%) were more synchronized to the control group in the intact condition relative to the scrambled condition.

### 3.2 Neural synchronization during naturalistic auditory stimulation

We applied the same CorrCA procedure to the EEG data from 15 different healthy controls while they listened to the intact version of *“Taken”*. The topography of the intact component showed a posterior negativity and widespread frontal positivity that was spatially analogous to the intact component from “*Bang! You’re Dead*” (Fig. 2A). However, this component was much less stable across leave-one-out subsets (spatial correlations, *r* > 0.67). Nevertheless, group-level mean ISCs (*M* = 0.019, *SD* = 0.009), remained significant, *t*(14) = 8.417, *p* = 7.55e-7, though reduced compared to “*Bang! You’re Dead*”, likely owing to the unimodal nature of the clip.

The group-level temporal ISCs showed that participants’ EEG activity was significantly synchronized throughout 15.79% of the audio and that these periods of synchronization were significantly correlated with its suspense ratings, *r* = 0.186, *p* = 2.00e-1, BF_10_ = 1.245. Like “*Bang! You’re Dead*”, this result indicated that the EEG activity was maximally synchronized at the group level during the most suspenseful points of the audio clip from “*Taken*”.

For the scrambled version of *“Taken”*, the CorrCA produced a component that differed considerably from the intact version and from either of the components calculated on the “*Bang! You’re Dead*” data (see SI Fig. 3 for topographies). This component was the least consistent between leave-one-out subsets (spatial correlations, minimum *r* = −0.44), though group-level mean ISCs (*M* = 0.016, *SD* = 0.009) were significant, *t*(14) = 7.073, *p* = 2.00e-1. However, like “*Bang! You’re Dead*”, the recalculation of the group-level ISCs after back-projection revealed that the neural activity underlying these components differed between conditions; we found significant reductions in ISCs for the intact condition after back-projecting the scrambled component, *t*(14) = 6.901, *p* = 7.28e-6 and, likewise, for the scrambled condition after back-projecting the intact component, *t*(14) = 5.612, *p* = 6.40e-5. Like “*Bang! You’re Dead*”, a 2x2 factorial ANOVA revealed a main effect of projection type, *F*(1,14) = 73.12, *p* = 6.30e-7, but no significant interaction between the variable, *F*(1,14) = 2.04, *p* = 1.75e-1 (SI Fig. 1B.)

**Fig. 3:**
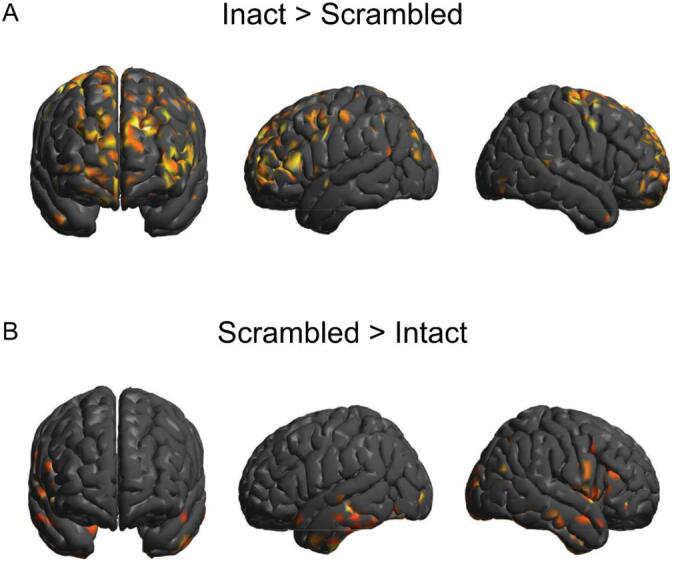
Source reconstruction of the top CorrCA components for the intact and scrambled versions of “*Taken*”. **A)** Source activations that were significantly correlated with the component time course from the intact version of “*Taken*” contrasted against the activity from the scrambled condition (intact > scrambled). **B)** Source activations that were significantly correlated with the component time course from the scrambled version of “*Taken*” contrasted against the activity from the intact condition (intact < scrambled).

For the group-level temporal ISCs, we found that participants neural activity synchronized during only 9.87% of the scrambled version of “*Taken*”. Moreover, like the scrambled version of “*Bang! You’re Dead*”, the temporal ISCs did not correlate with the suspense ratings for “*Taken*”, *r* = 0.107. *p =* 2.00e-1, BF_10_ = 0.233, suggesting again that synchronization among participants in this condition was not plot-based (SI Fig. 4 A, B).

With the component from the intact version of “*Taken*”, we calculated the ISCs between ten patients with DOC and the healthy control group. Here, we found that 30% of patients produced EEG activity that was significantly correlated with controls during this movie (Fig. 2B). Of these patients, one was diagnosed with Locked-in Syndrome (Table 1), while the remaining patients met the behavioural criteria for a vegetative state diagnosis. EEG data from the scrambled audio condition was available for 9 of the 10 patients who listened to the intact version of “*Taken*”. As was the case with “*Bang! You’re Dead*”, we found synchronization was reduced in the scrambled version; that is, most patients (78%) were more synchronized to the control group while they watched the interact version relative to the scrambled version. Nevertheless, following the same back-projection procedure, we did not find significant correlations between the EEG activity of any DOC patients and healthy controls during this condition.

### 3.3 Source localization

Finally, we performed a source localization analysis on the healthy control data from both versions of “*Taken*” to investigate the neural generators of the components. Paired *t* contrasts were calculated on the cortical activations that most strongly correlated with the time courses of the intact and scrambled components. This revealed a significant difference in overall activation between movie conditions (SPM paired *t* contrasts at *p* < 0.05) The intact > scrambled contrast showed greater bilateral activation over frontal and parietal regions (Fig. 3A), whereas the scrambled > intact contrast revealed only sparse activation over anterior regions of the inferior and middle temporal cortices (Fig. 3B). Despite the exploratory nature of this analysis, the differences in cortical activity between movie conditions closely resembled previous findings in fMRI (Naci et al., 2015, Naci et al., 2014).

To ensure that these results did not simply reflect differences in the auditory characteristics between the intact and scrambled movie, we performed a follow-up analysis to identify the brain areas associated with processing the low-level auditory properties of “*Taken*”. Specifically, we contrasted the cortical response to the physical features of the intact audio (i.e., its pitch, timbre, and loudness—captured by its auditory envelope) to the activity elicited by the full audio clip (containing speech and the plot). We performed paired *t* contrasts between the intact audio > auditory envelope and auditory envelope > intact audio and found that there was a significant difference between the overall source activations for each condition, *t*(14) = 3.79, *p* = 1.00e-3. Moreover, the difference maps between contrasts bore a considerable resemblance to the intact > scrambled localization analysis. From these analyses, we, therefore, concluded that neither the auditory envelope of the intact version of “*Taken*”, nor the perceptual features of the scrambled movie generated the frontoparietal activation observed during the intact audio condition.

## 4 Discussion

Overall, we found that the EEG responses of 38% of DOC patients (four vegetative, one Locked-in) in this cohort were significantly correlated to those of healthy controls during at least one of our movie clips. This result suggests that these patients may have retained or recovered some of the “executive” faculties necessary for processing the plot of the movie stimuli we used (Naci et al., 2015, Naci et al., 2014). This percentage (38%), is higher than previous studies that have used neuroimaging and covert command-following (14% of vegetative patients, 32% of minimally conscious patients; Kondziella et al., 2016). This potentially speaks to the simplicity of our movie paradigm, as well as the inherent ease with which we attend to engaging movie stimuli (Dmochowski et al., 2014, Hasson et al., 2004, Ki et al., 2016, Naci et al., 2015). However, the percentages reported here reflect findings across a small cohort of DOC patients and should be interpreted with caution when compared to the larger body of literature. Repeat testing and validation among a larger sample of DOC patients would be needed before the proportion of cognitively capable DOC patients reported in this study could be appropriately applied to the population as a whole.

Although it is challenging to infer the cognitive states of DOC patients from these results alone, significant correlations in neural activity between these patients and healthy controls during our movie tasks suggest that they may have been having a comparable experience of the plot for a number of reasons. In particular, the results from our analysis of our healthy control data align with previous studies (in both component topography and the magnitude of ISCs) that used CorrCA to examine the neural processes of engagement associated with movie-watching (Cohen and Parra, 2016, Dmochowski et al., 2012, Ki et al., 2016, Poulsen et al., 2017) and, importantly, with those of a recent investigation of the electrophysiological markers of auditory attention in DOC patients (Iotzov et al., 2017). In that study, Iotzov et al. recorded EEG activity from patients with DOC while they listened to a spoken narrative and compared their responses to that of healthy controls on three components derived from a CorrCA. At the group level, Iotzov et al. observed a significant reduction in ISCs for DOC patients compared to control across all three components and found some evidence that the magnitude of ISCs corresponded to clinical diagnosis. We also performed three additional analyses to disentangle the ISCs generated from the sensory properties of the movies from those driven by the plot. First, we back-projected the components from the intact and scrambled movies onto the EEG data from the other movie condition (intact onto scrambled, scrambled onto intact). This created a spatial filter that isolated the neural signal of the intact component in the scrambled EEG data and vice versa. Had the components for each condition captured the same neural processes, we would have expected no change in the ISCs. However, using this method, we found consistent and significant decreases in mean ISCs for both “*Bang! You’re Dead*” and “*Taken*”. The reduction in mean ISCs demonstrated that the components from each movie condition encompassed different neural processes.

We then compared the time course of inter-subject synchronization, computed using temporal ISCs, with the suspense ratings for each movie. We found that participants were maximally synchronized during time windows that corresponded to the most suspenseful periods of each movie but only during the intact (and not the scrambled) conditions. This provided further evidence that the components calculated for the intact version of the movies represented brain activity associated with executive processing necessary to track the narrative, rather than the sensory properties of the movies. Lastly, the source reconstruction of the components from “*Taken*” revealed a clear separation between the brain regions involved in processing the intact and scrambled versions of the movie. That is, the intact component was localized primarily to the frontoparietal cortices, whereas the scrambled component activity was localized largely to temporal auditory regions, aligning closely to results shown in fMRI (Hasson et al., 2004, Naci et al., 2015, Naci et al., 2014). This suggests patients are recruiting the same set of executive processes (i.e., attention, language processing, memory, and theory of mind; Naci et al., 2014, Cohen and Parra, 2016, Ki et al., 2016) that are essential for plot following.

How do we know that synchronization between DOC patients and healthy controls is not the result of some kind of automatic or unconscious processing? While previous studies on the neural effects of anesthesia have shown that inter-subject neural synchronization can occur in low-level brain areas in the absence of awareness (Naci et al., 2018), we contend that automatic or unconscious processing alone cannot explain significant ISCs during the intact movies in our study. Indeed, the source results for the intact and scrambled “*Taken*” components share the same distinct activation patterns found in similar fMRI paradigms (Naci et al., 2015, Naci et al., 2014). Frontoparietal synchronization has been shown to correlate strongly with higher-order elements of movie stimuli, like its plot, which cannot be processed unconsciously (Naci et al., 2018). Furthermore, if ISCs during the intact movie conditions were primarily sensory-driven, we would expect the components to index the same neural processes as the scrambled components. Our back-projection analysis determined that this was not the case, despite the sensory properties of the stimuli being largely the same between conditions. Finally, neural synchronization is not a natural state of the brain; it does not occur when participants are at rest (Hasson et al., 2004, Naci et al., 2014) and is much weaker in the absence of focused attention (Ki et al., 2016) or during non-engaging stimuli (Dmochowski et al., 2012, Hasson et al., 2010).

There are some peculiarities in our patient results that should be addressed. First, the majority of patients who had significant ISCs with healthy controls were behaviourally vegetative, not minimally conscious. One factor that may account for this result relates to data quality; EEG is very susceptible to movement artifacts, which may have been more prevalent for the minimally conscious patients (who are more likely to move overall), potentially impacting their ISCs with the healthy group. Similarly, some percentage of vegetative patients are likely to be covertly aware but simply cannot express this through their behaviour, whereas minimally conscious patients are, as their diagnosis suggests, minimally conscious and therefore have limited cognitive, as well as behavioural capacities. As a result, patients who are behaviourally vegetative but fully aware would be expected to process movie stimuli similarly to healthy controls, while patients who are minimally conscious may experience more difficulties, lowering their overall ISCs with controls.

A second notable finding comes from the patient ISCs during the scrambled conditions; the two patients who were significantly synchronized with the control group during the scrambled version of “*Bang! You’re Dead*” were not synchronized with the control group during the intact condition. A possible explanation for these results is that the two patients who were synchronized with controls during the scrambled version retained some cognitive or attentional resources and were minimally engaged while it played. This is possible because the scrambled version of “*Bang! You’re Dead*” contained some residual structure. However, the neural activity from these patients was not significantly synchronized during in the intact version of the movie, perhaps due to fatigue (the intact movie was presented after the scrambled version), or disinterest. This itself is not unusual; even among the healthy control group, one participant whose EEG was synchronized with the rest of the control group during the scrambled version of the movie was not significantly synchronized during the intact version. Such findings speak to the inherent variability associated with measures designed to assess individual cases. This provides added motivation for evaluating the reliability of this method for determining residual cognitive processing in the patients with DOC, ideally by conducting longitudinal studies whereby repeated measures are taken.

Overall, 38% of patients tested were significantly synchronized with healthy controls during either “*Bang! You’re Dead*” or “*Taken*”. However, among these patients, only one (Patient 10, see Table 1) showed significant ISCs with controls during both movies (of the nine who were tested with both). While significant synchronization during both movies provides the strongest evidence of residual processing, inconsistencies in ISCs between movie types for most patients underscores the need for a holistic testing approach that employs multiple tasks to identify covert cognitive processing in this population (Engemann et al., 2018, Gibson et al., 2014, Kirschner et al., 2015, Sitt et al., 2014). Individual patients with DOC likely have marked differences in sensory and cognitive function, and brain-based assessments should be designed with this in mind.

The results of this study set the stage for developing sensitive and reliable brain-based assessments of covert cognitive processing and, potentially, awareness in patients with DOC—ideally, ones that can be administered easily in clinical settings. The paradigm presented here moves one step closer to achieving this goal. By developing a bedside EEG movie task (Naci et al., 2015, Naci et al., 2014), we were able to quantify a neural index of cognitive processing while simultaneously minimizing the physical burden to patients incurred during fMRI testing. Likewise, the majority of EEG tasks used to assess cognitive function and awareness in DOC patients to date have done so by examining changes in neural activity that are either elicited automatically (e.g., event-related potentials; Kotchoubey et al., 2005) or depend upon active responding (Cruse et al., 2012, Cruse et al., 2011). In both contexts, these paradigms are often contrived or unnatural, making an already difficult task even more challenging. Furthermore, the event-related approaches routinely used for clinical neurological assessments require hundreds of trials to open a brief window into the sensory and cognitive function of DOC patients; whereas, our method was specifically developed to work with a single sample of continuous EEG, recorded during a short naturalistic movie task, to assess covert cognition in individual patients with DOC.

For any task to be included in the standard clinical assessment repertoire, it must be rapid and allow for individual assessments of cognition at the bedside without the need for complex tasks or instructions. Our paradigm meets all of those requirements. Taking cues from continuous clinical monitoring and brain-computer interfaces (Abdalmalak et al., 2017, Chatelle et al., 2012, Laureys et al., 2005, Naci et al., 2012), the future of the CorrCA method could allow for examination of moment-to-moment ISCs between DOC patients and controls during movie tasks, further supplementing behavioural measures of awareness at the bedside.

## CRediT authorship contribution statement

**Geoffrey Laforge:** Conceptualization, Data curation, Formal analysis, Investigation, Methodology, Software, Validation, Visualization, writing. **Laura E. Gonzalez-Lara:** Data curation, Investigation, writing. **Adrian M. Owen:** Conceptualization, Funding acquisition, Investigation, Methodology, Project administration, Resources, Supervision, Visualization, writing. **Bobby Stojanoski:** Conceptualization, Data curation, Formal analysis, Investigation, Methodology, Project administration, Resources, Software, Supervision, Validation, Visualization, writing.

## Funding

This work was supported by the 10.13039/501100002784Canada Excellence Research Chairs Program (Grant # 215063), 10.13039/501100000024Canadian Institutes of Health Research (Grant # 300292), 10.13039/100007631Canadian Institute for Advanced Research (CIFAR). The sponsor of the study had no role in study design, data collection, data analysis, data interpretation, or writing of the report. The authors had full access to all the data in the study and had final responsibility for the decision to submit for publication.

## Declaration of Competing Interest

The authors declare that they have no known competing financial interests or personal relationships that could have appeared to influence the work reported in this paper.
