# Supplementary material for: Individualized assessment of residual cognition in patients with disorders of consciousness
Source: Neuroimage Clin. 2020 Oct 20;28:102472. doi: 10.1016/j.nicl.2020.102472 (PMC7652775; doi:10.1016/j.nicl.2020.102472)
Supplement: Supplementary data 1 [file mmc1.docx]

**Individualized Assessment of Awareness in Patients with Disorders of Consciousness: Supplementary Material**

Geoffrey Laforge M.Sc.^a^, Laura E. Gonzalez-Lara Ph.D.^a^, Adrian M. Owen Ph.D.^a,b,c^, & Bobby Stojanoski Ph.D.^a,b^

^a^ The Brain and Mind Institute, The University of Western Ontario, London ON, N6A 5B7, Canada.

^b^ The Department of Psychology, The University of Western Ontario, London ON, N6A 5B7, Canada.

^c^ The Department of Physiology and Pharmacology, The University of Western Ontario, London ON, N6A 5B7, Canada.

Corresponding author: Geoffrey Laforge

Corresponding author’s address: 1151 Richmond St, London, ON. Canada N6A 3K7

Corresponding author’s phone and fax: (1) 519-661-2111, ext. 84672

Corresponding author’s e-mail address: glaforge@uwo.ca


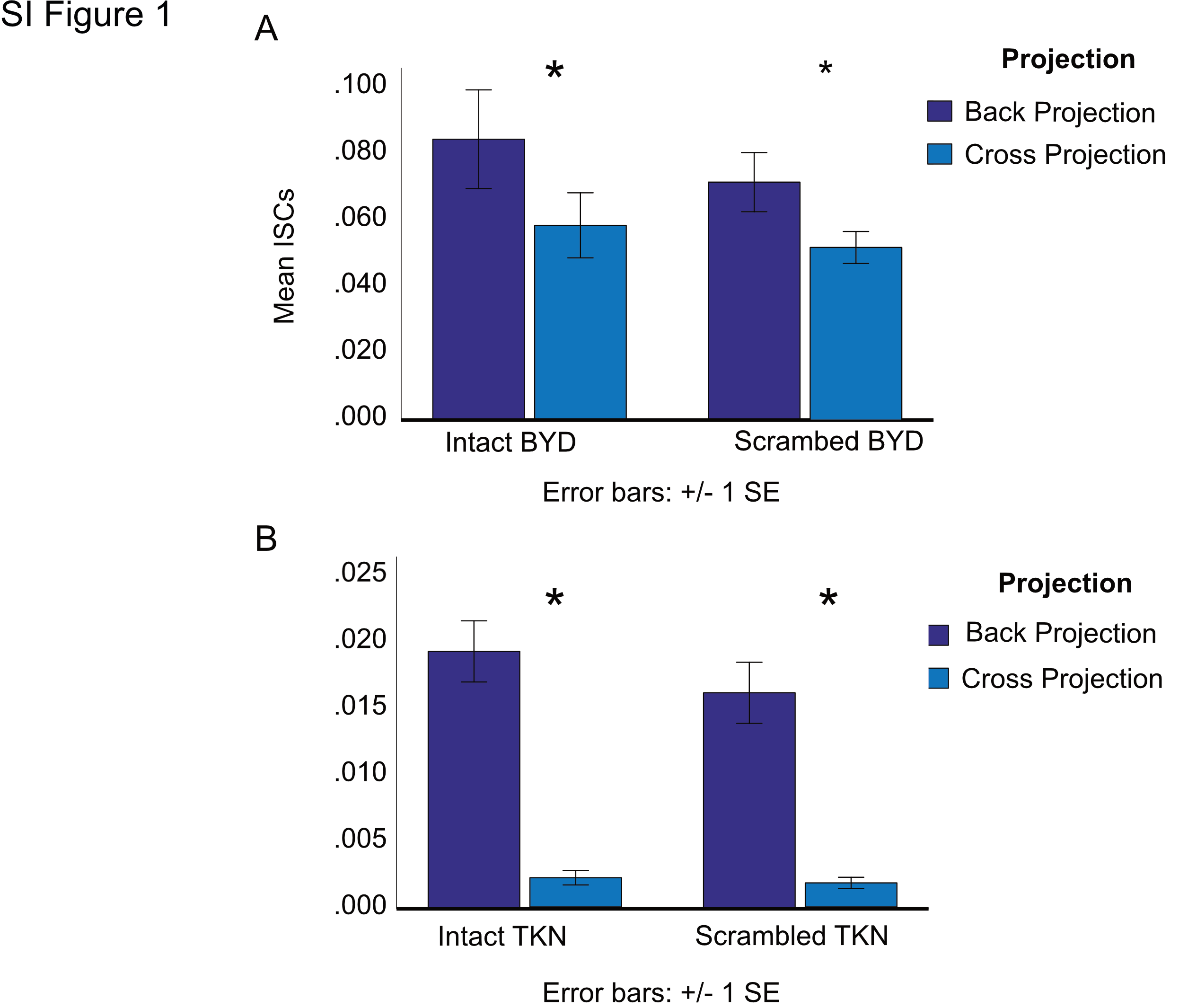


**Supplementary Figure 1.** Factorial ANOVA results for the cross-projection analysis. We performed a 2x2 factorial ANOVA to investigate potential interaction effects of our cross-projection analysis, where we computed inter-subject correlations (ISCs) for the data in one condition using the components from the other (e.g., computing ISCs for the scrambled audio using the intact audio component). **A)** The 2x2 factorial ANOVA revealed a significant main effect of projection type, *F*(1,12) = 20.83, *p* = 7e-4, but no interaction, *F*(1,12) = 0.73, *p* = 0.41, for the intact or scrambled versions of “*Bang! You’re Dead*”. **B)** Similarly, only the main effect of projection type, *F*(1,14) = 73.12, *p* = 6.3e-7, was significant during the intact and scrambled version of “*Taken*”.


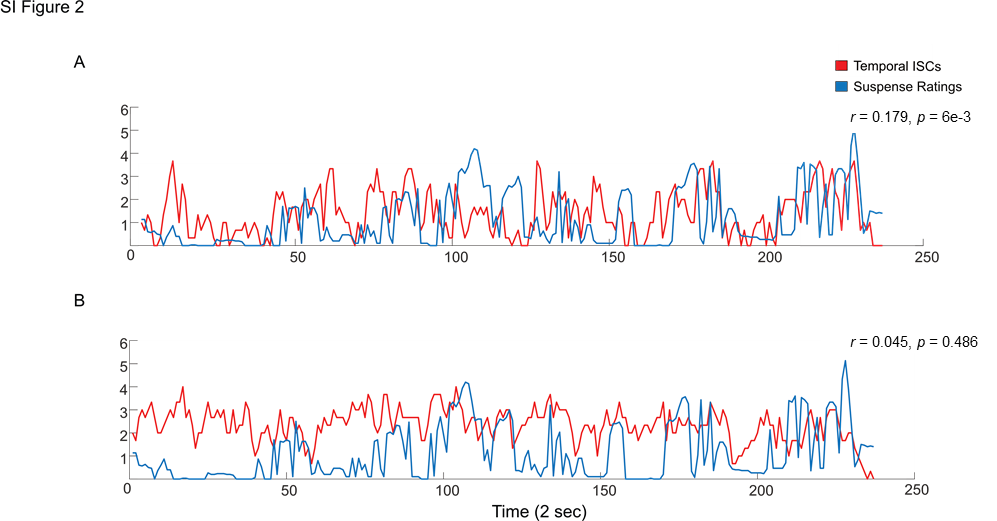


**Supplementary Figure 2.** Correlations between temporal ISCs and suspense ratings for the intact and scrambled versions of “*Bang! You’re Dead*”. **A)** The time course of temporal ISCs (red) for the intact version of “*Bang! You’re Dead*” were significantly correlated to the suspense ratings for the movie (blue), *r* = 0.179, *p* = 6e-3. **B)** We did not find a significant correlation between the temporal ISCs (red) and the suspense ratings (blue) for the scrambled version of “*Bang! You’re Dead*”, *r* = 0.045, *p* = 0.486. *Note*: Suspense ratings were scaled down to improve visualization, but correlations were computed on unscaled values.


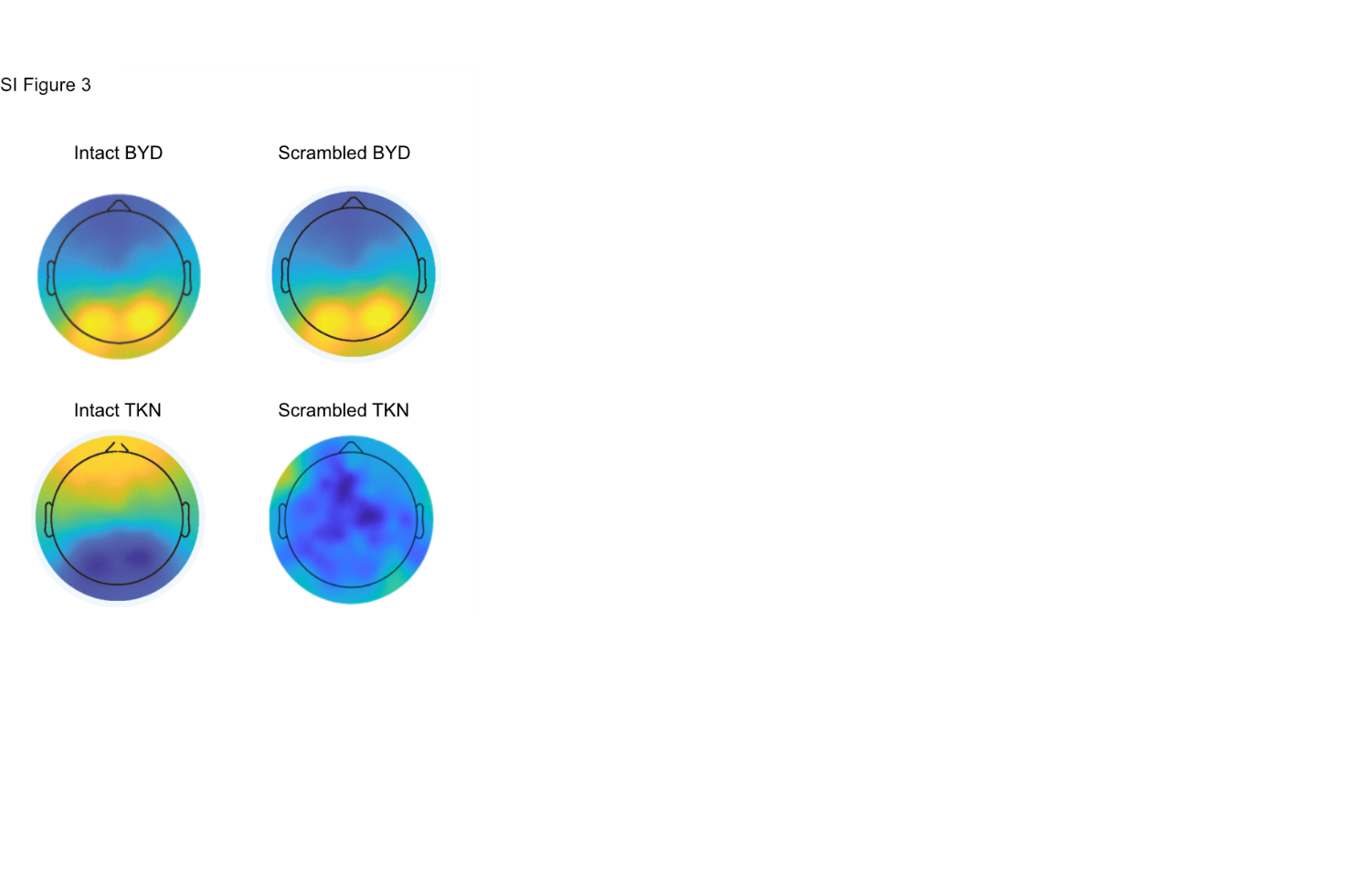


**Supplementary Figure 3.** CorrCA component topographies from healthy controls during the intact and scrambled versions of “*Bang! You’re Dead*” and “*Taken*”. The component topography calculated on the EEG data from the intact version of “*Bang! You’re Dead*” (left, top) showed a considerable degree of similarity with the scrambled component (right, top). However, the component from the intact version of “*Taken*” (left, bottom) was highly dissimilar to the scrambled component (right, bottom).


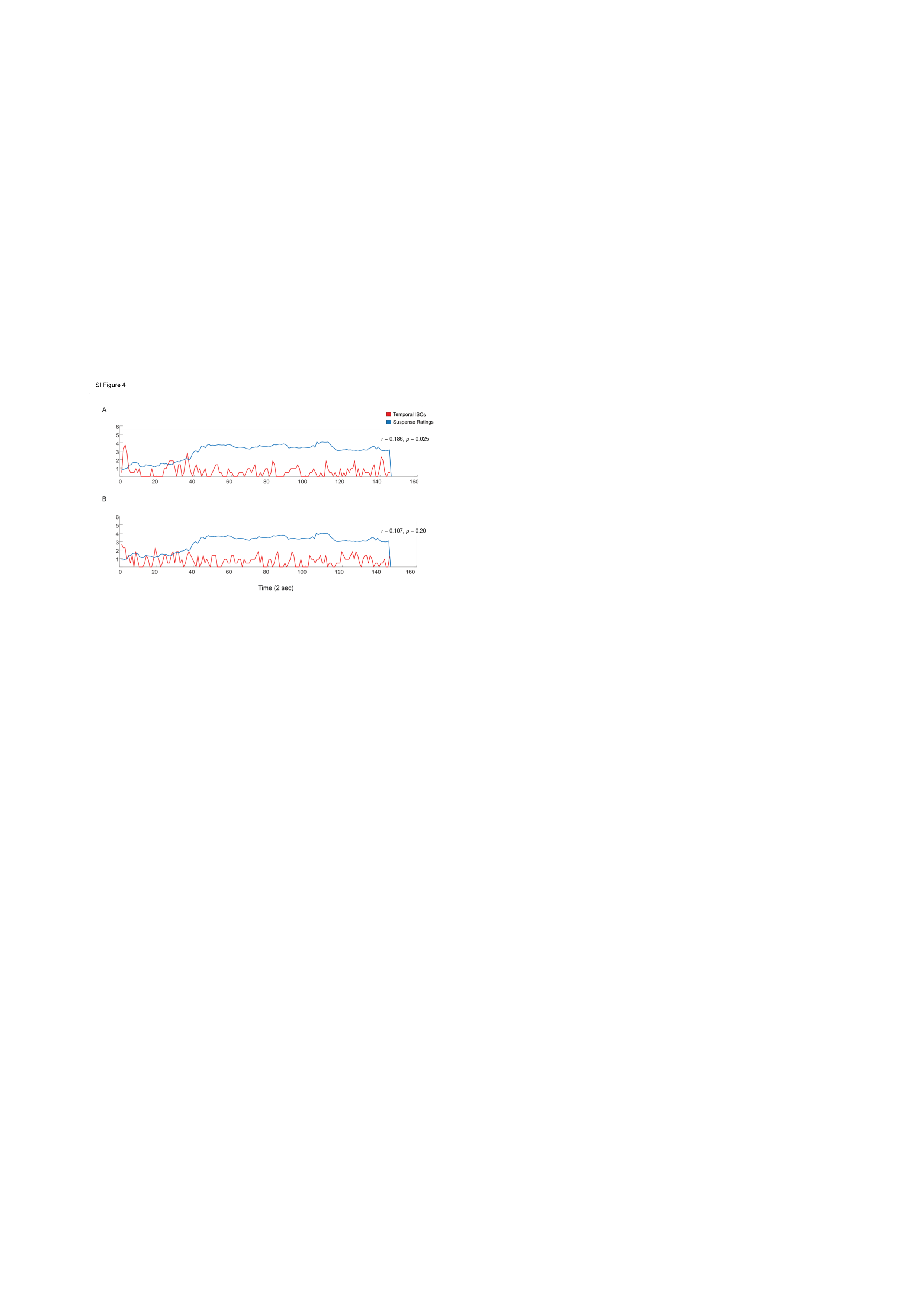


**Supplementary Figure 4.** Correlations between temporal ISCs and suspense ratings for the intact and scrambled versions of “*Taken*”. **A)** The time course of temporal ISCs (red) for the intact version of “*Taken* were significantly correlated to the suspense ratings for the movie (blue), *r* = 0.186, *p* = 0.025. **B)** Like *“Bang! You’re Dead*”, the correlation between the temporal ISCs (red) for the scrambled version of “*Taken*” and its suspense ratings (blue) were not statistically significant, *r* = 0.107, *p* = 0.20.


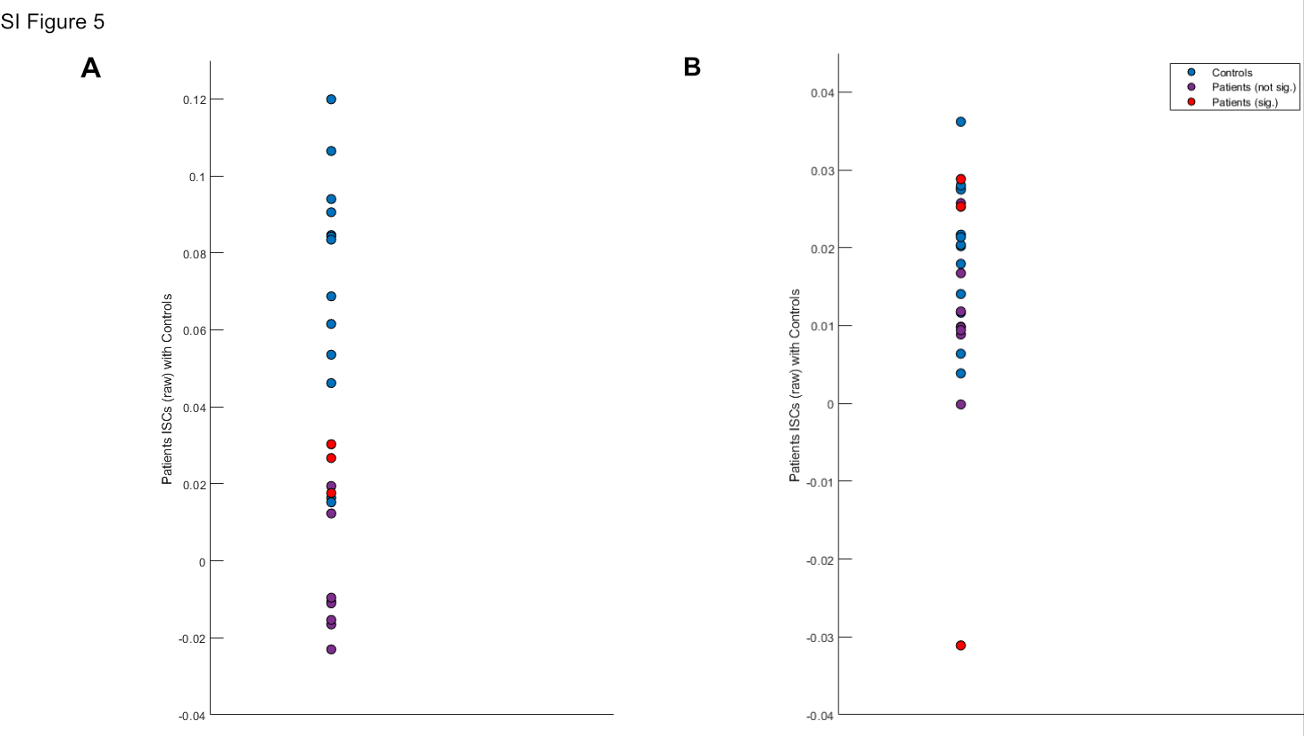


**Supplementary Figure 5.** Raw ISCs between patients and healthy controls for the intact version of *“Bang! You’re Dead*” and “*Taken*”. **A)** Raw ISCs between patients (not significant, purple; significant, red) and healthy controls for “*Bang! You’re Dead*”. **B)** Raw ISCs between patients (not significant, purple; significant, red) and healthy controls for “*Taken*”. Absolute ISCs were reported in the manuscript to account for individual differences in dipole orientation (polarity) relative to the group-level component projection.
